# Distinct CD4^+^CD8^+^ Double-Positive T Cells in the Blood and Liver of Patients during Chronic Hepatitis B and C

**DOI:** 10.1371/journal.pone.0020145

**Published:** 2011-05-25

**Authors:** Michelina Nascimbeni, Stanislas Pol, Bertrand Saunier

**Affiliations:** 1 Faculty of Medicine, Paris-Descartes University, Paris, France; 2 Institut Cochin, UMR8104 of the Centre National de la Recherche Scientific (CNRS), Paris, France; 3 U1016 of the Institut National de la Santé et de la Recherche Médicale (Inserm), Paris, France; 4 Hepatology Unit of Cochin Hospital, Assistance Publique-Hôpitaux de Paris (AP-HP), Paris, France; Centre de Recherche Public de la Santé (CRP-Santé), Luxembourg

## Abstract

CD4^+^ and CD8^+^ T cells, the main effectors of adaptive cellular immune responses, differentiate from immature, non-functional CD4^+^CD8^+^ double-positive T (DPT) cells in the thymus. Increased proportions of circulating DPT lymphocytes have been observed during acute viral infections; in chronic viral diseases, the role and repartition of extra-thymic DPT cells remain largely uncharacterized. We performed a phenotypic analysis of DPT cells in blood and liver from patients chronically infected by hepatitis C (HCV) or B (HBV) viruses. The highest percentages of DPT cells, predominantly CD4^high^CD8^low^, were observed in patients infected by HCV, while HBV-infected patients mostly displayed CD4^low^CD8^high^ and CD4^high^CD8^high^ DPT cells. All proportions of DPT cells were higher in liver than in blood with, for each subpopulation referred to above, a correlation between their frequencies in these two compartments. In HCV patients, intra-hepatic DPT cells displayed more heterogeneous activation, differentiation and memory phenotypes than in the blood; most of them expressed CD1a, a marker of T cell development in the thymus. *Ex vivo*, the inoculation of liver slices with HCV produced in cell culture was accompanied by a disappearance of CD8^high^ cells, suggesting a direct effect of the virus on the phenotype of DPT cells in the liver. Our results suggest that, in half of the patients, chronic HCV infection promotes the production of DPT cells, perhaps by their re-induction in the thymus and selection in the liver.

## Introduction

CD4^+^ and CD8^+^ T cells are the main effectors of the adaptive cellular immune responses, and are required to clear viral infections [1]. These cells derive from precursors migrating from the bone marrow to the thymus, where they express rearranged T cell receptors (TCR) and CD3 together with both CD4 and CD8 co-receptors, from which CD4^+^ or CD8^+^ single positive naïve T cells are selected [2]. A small proportion of circulating CD4^+^CD8^+^ double-positive T (DPT) cells has been described in humans [3], as well as in animal models [4]–[7]. A few of them are immature T cells escaping from the thymus [8]. Others may be terminally differentiated cells re-expressing the second TCR co-receptor before their death [2]. More often, DPT cells are functional effector/memory T cells specific for antigens from a variety of viral pathogens [9], [10]. Their proportion can increase from about 1% up to 20% of circulating lymphocytes during infections by human immunodeficiency virus (HIV) or Epstein-Barr virus [11], [12]. Extra-thymic DPT lymphocytes are probably not restricted to viral infections, since they have also been found associated with malignant tumors [13], [14] or in target organs of autoimmune conditions [2], [15]. The roles played by extra-thymic DPT cells are still largely unknown, and their repartition only partially characterized.

DPT cells have been observed at low frequencies in the blood of patients chronically infected by HCV [9]. This virus establishes a persistent infection in the liver of about 75% of the patients; it is still not understood why the immune system fails to clear HCV in most instances. During the acute phase of the experimental infection of a chimpanzee with HCV (the only animal model that develops chronic infection, as in humans), the frequencies of both circulating and intra-hepatic DPT cells increased [9]. Correlation of the proportions of these cells with serum viral loads suggested a link between DPT cells and control of HCV infection. However, this result was obtained in an animal that spontaneously resolved its HCV infection, and has not been compared to those of animals becoming chronically infected. In addition, no data is available regarding DPT cells in the liver of HCV-infected patients. In this study, we sought to concomitantly determine the patterns of DPT cells in peripheral blood and liver of chronically HCV-infected patients. We compared these patterns with those of patients presenting with chronic infections of the liver by HBV.

## Materials and Methods

### Patients and human tissues, sample preparation

Twenty-seven chronically HCV mono-infected (57±10 year old), eight HBV mono-infected patients (40±9), six HCV-HIV co-infected (50±13), three non-infected patients and two healthy individuals were included in this study. All subjects were followed in the Liver Unit of Cochin Hospital (AP-HP, Paris, France); Institutional review board of Cochin Hospital approved the study protocol.

Paired blood samples and liver biopsies were obtained from all patients who had an indication for a liver biopsy to follow up or stage their liver condition; large piece of the biopsies were fixed and examined by the Pathology Unit of Cochin Hospital; stage of fibrosis was determined with certainty in twenty HCV mono-infected (5 F1, 8 F2, 5 F3 and 2 F4) and seven HBV mono-infected (2 F0, 3 F1, 2 F2 and 1 F3) patients. Part of the fresh liver biopsies (>1-cm long, 1-mm diameter) were mechanically disrupted on a 100 µm-pore mesh in 3 ml of non-supplemented RPMI culture medium; freshly isolated liver infiltrating lymphocytes (LILs) were centrifuged, resuspended in phosphate buffer saline (PBS), pH = 7.4, containing 2% fetal calf serum (FCS), and immediately stained for phenotyping analysis. Peripheral blood mononuclear cells (PBMCs) were isolated from blood by centrifugation over a Ficoll gradient, washed twice and re-suspended in PBS supplemented with 2% FCS. For one non-infected and one HCV-infected patients liver biopsies were snap frozen for *in situ* analysis.

### Flow cytometry

Freshly isolated paired PBMCs and LILs were incubated with 4 µl of the BD Multitest 6-Color TBNK Reagent (BD Biosciences) for 30 minutes at 4°C in the dark. The cells were washed, incubated with paraformaldehyde 2% in PBS, harvested on a FACSCanto or a LSRII (Becton Dickinson) and analyzed with Diva or FlowJo softwares. Cell death evaluated with the Live/Dead Fixable Blue Dead-Cell-Stain Kit (Molecular Probes, Invitrogen) as indicated by the manufacturer, was always lower than 5% of DPT cells. In average, the total number of events acquired was 10^6^; a result below 100 DPT cells was considered as not meaningful and plotted as zero percent.

### *In situ* immunofluorescence

Snap-frozen liver biopsies were cut in 10-µm serial sections using a cryotome (Leica CM), fixed in acetone, incubated with PBS/5% BSA/5% AB serum for 30 min, then with primary antibodies (CD4 (Novacastra), CD8, CD3 (Abcam); 1/100 dilution each) for 1h30. After two washes in PBS containing 0.5% Tween 20, sections were incubated with 1/200 diluted Alexa-Fluor-488- and -546-coupled secondary antibodies for an hour, washed, fixed in paraformaldehyde 2% in PBS, counterstained with Hoechst (1 µg/ml) and incubated with MEM Essential Amino Acids for 20 minutes. Slides were mounted with Fluoromount-G (Southern Biotech). Sections were analyzed with an Axiovert-100M Zeiss microscope equipped with an Orca ER camera (−20°C, pixels 1344/1024, Hamamatsu). Photographs were colorized with Image J 1.38. The fluorescence intensities of the red and green channels were obtained with the Plot Profile function of the software.

### Human liver slices

Macroscopically non-tumoral/non-pathological liver tissue was collected from a HCV-, HIV- and HBV-negative patient undergoing surgery for liver tumor resection. Serial 350 µm-thick slices were obtained by cutting the liver sample in ice cold PBS using a Vibratome (Leica, Heidelberg, Germany) and placed onto Millipore filters in Dulbecco's modified Eagle's medium with glutamine containing 10% fetal calf serum, MEM non essential amino acids, 25 mM HEPES and penicillin-streptomycin (Life Technologies, MD), then incubated at 37°C in an H_2_O-saturated atmosphere comprised of 95% air-5% CO_2_.

### Production of HCV particles in cell culture

HCVcc (JFH-1 strain of genotype 2a) was produced in HuH-7.5 cells, as previously described [16]. Briefly, culture supernatants were harvested, clarified by low-speed centrifugation to remove cell debris, filtered through 0.45 µm PVDF membranes, concentrated using Vivaspin filters (MWCO = 1,000,000 daltons), and kept frozen at −80°C until used.

### Statistical analyses

Pearson's correlation between the proportions of DPT cell subpopulations in blood and liver for each patient was tested using a t-test; the ratios of CD4^high^CD8^low^ over CD4^low^CD8^high^ DPT cells were compared using a Mann-Whitney (non-parametric) test.

### Ethics Statement

The patients' informed consent was obtained in writing prior to collecting samples during routine medical visits, and in compliance with the standard Ethical Guidelines of the Institutional Review Board of Cochin Hospital (Paris) who approved the study.

## Results

### HCV-infected patients have often a high proportion of DPT cells in both blood and liver

**Figure 1:**
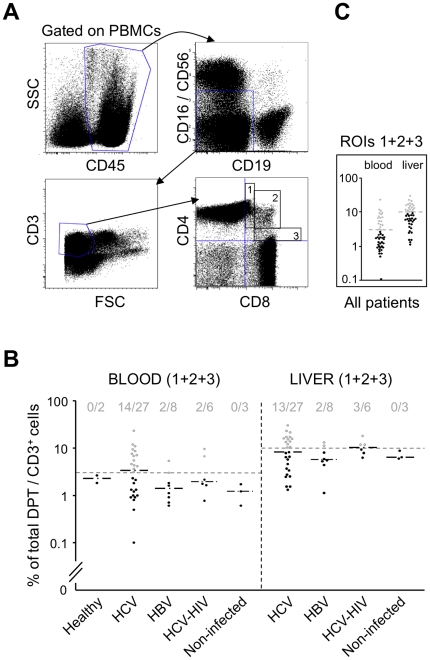
Proportions of DPT cells in the blood and liver of chronically HCV- or HBV-infected, HCV-HIV co-infected and non-infected patients. **A,** Representative dot plot of PBMCs labeled with the BD Multitest 6-Color TBNK Reagent: CD45^+^CD16^−^CD56^−^ CD19^−^CD3^+^ PBMCs were analyzed for CD4 and CD8 expressions; viability of the CD4^+^CD8^+^ cells was separately assessed with Live/Dead Cell Viability Assay (>95%). **B,** Proportions of total CD4^+^CD8^+^CD3^+^ (DPT) cells in peripheral blood and liver biopsy for each patient of the different groups. In grey, the experimental points with values above the thresholds (dashed lines) determined in (**C**), with the corresponding numbers of patients at the top (above threshold/total). **C,** Determination of the thresholds: 3% in the blood and 10% in the liver.

To analyze DPT cells, freshly isolated PBMCs and LILs were stained with fluorochrome-coupled antibodies recognizing immune cell surface markers. Within the CD45^+^ cell population, and after exclusion of CD16^+^ CD56^+^ NK (or NKT) cells and CD19^+^ B cells, the CD3^+^ T cells were analyzed for CD4 and CD8 expressions (Figure 1A). The bottom right panel of Figure 1A further depicts how we determined the proportion of total DPT cells —regions of interest (ROIs) 1, 2 and 3 in the upper right quadrant— in blood and liver from chronically HCV-infected patients; the results are shown in Figure 1B. The average proportion of these cells in the liver was more than 2-fold (mean ± SD = 10.1±7.5%; median = 8.2%) that in the blood (4.6±5%; 3.1%), which suggests that DPT cells are sequestered in the liver or locally induced. However, since the liver of uninfected patients also contained a higher proportion of DPT cells (Figure 1B), the difference of their proportions between these two compartments was not linked to HCV infection.

We found higher proportions of circulating DPT cells than in a previous study [9], which could relate to their more stringent phenotyping (cf. Materials and Methods) or other methodological factors, such as ethnicity (not accounted for in France) and way of life (obesity, alcohol consumption, etc…), or viral genotype: the present group included mixed HCV genotypes (with a majority of 1b), whereas the previous only included genotype 1 (mostly 1a) [9]. Another factor could be the stage of clinical progression. In five patients presenting with acute HCV infections (<4 months), we observed proportions of circulating DPT cells ranging between 0.05 and 1.3%, similarly to uninfected patients. However, this result might not reflect intra-hepatic DPT cells, if not re-circulating. In addition, proportions of circulating DPT cells were not determined during the very first weeks of HCV primo-infection; as spontaneous clearance of HCV infection occurs much more frequently in the chimpanzee than in acute patients, we do not know what the outcome of the five patients in our study would have been in the absence of treatment. It nevertheless indicates that proportions of circulating DPT cells vary with the step of HCV infection. Thus, although the stages of chronic HCV infections (early vs. late) at which patients were included have not been determined, they could explain differences between these two studies.

### HCV-infected patients have a higher proportion of DPT cells than HBV-infected patients

In all groups, frequencies of DPT cells in the liver were higher than in the blood. The numbers of patients were too small in some groups to meaningfully perform multiple comparisons; their values are only provided as an indication. In the blood, the proportion of DPT cells in HBV mono-infected patients (1.9±1.6%; 1.4%) were not very different from those in non-infected patients (1.2±0.6%; 1.2%) and healthy controls (2.2±0.6%; 2.2%), but lower than in HCV-infected patients (4.6±5%; 3.1%), or HCV-HIV co-infected (4.5±3.8%; 1.9%). In the liver, the proportions of DPT cells in HBV mono-infected (6.7±3.8%; 5.6%) and non-infected (6.9±1.5%; 6.3%) patients were also lower than in HCV-mono-infected (10.1±7.5%; 8.2%) or HCV-HIV co-infected (12.3±6.1%; 11.9%) patients. The percentages of DPT cells in the two groups of HCV and HBV mono-infected patients were compared using a non-parametric test (Mann-Whitney); the difference reached statistical significance in the blood (p<0.05), but not in the liver (p>0.15).

In all groups, the proportions of DPT cells were spread along a broad range of values with a bottleneck in the number of patients (Figure 1C). In the blood, it was observed for around 3% DPT cells. Albeit less obvious in the liver, based on the difference of proportions of DPT cells between blood and liver (cf. above), a three-time higher threshold was determined. Strikingly, almost all patients with proportions of DPT cells above the thresholds were HCV mono-infected (blood: 14/27; liver: 13/27) or HCV-HIV co-infected (blood: 2/6; liver: 3/6), except for two who were mono-infected by HBV (2/8 for both blood and liver) and had values close to the thresholds (Figure 1B). This suggested that about half of chronically HCV-infected patients had a higher proportion of DPT cells, both in blood and in liver, the bottleneck reflecting perhaps the existence of two types of patients, which could underlie a lack of power to reach statistical significance in the liver.

### Predominant CD4^high^CD8^low^ DPT cell subpopulation in chronically HCV-infected patients

In fact, three main phenotypes of DPT cells can be distinguished with flow cytometry analyses: CD4^high^CD8^low^, CD4^low^CD8^high^ and a less numerous subpopulation of CD4^high^CD8^high^ DPT cells: in Figure 1A (lower right panel) these populations respectively correspond to ROIs 1, 3 and 2 (upper right quadrant). The former are generally CD8alpha-alpha, and may correspond to terminally differentiated cells [2]; in fact, some of them are NKT cells [17], but those cells were excluded from our analysis (cf. above). CD4^low^CD8^high^ DPT cells are CD8alpha-beta [2], which has raised the hypothesis that those in the peripheral blood might be terminally differentiated cells generated during viral infections [9]. However, this idea has been questioned, in particular by the recent report that the proportion of circulating CD4^high^CD8^low^ T cells increased during HIV infections [18]. Therefore, CD4^high^CD8^low^ DPT cells, also observed within tumors [19], might instead be generated during chronic immune processes. We therefore reanalyzed the proportions of total DPT cells according to these three subpopulations, looking for the presence of pattern specificities, or lack thereof, in the blood and the liver of patients chronically infected by HCV or HBV.

**Figure 2:**
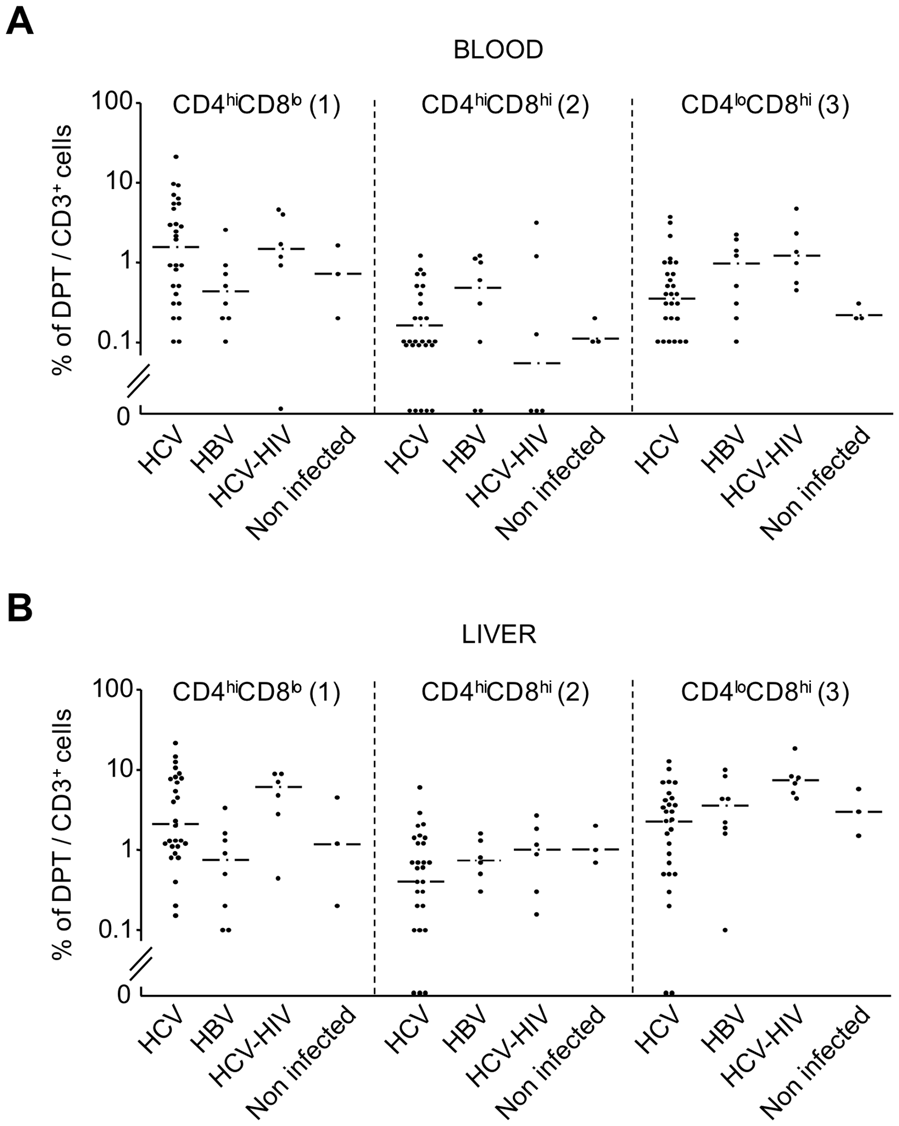
Proportions of CD4^high^CD8^low^, CD4^high^CD8^high^ and CD4^low^CD8^high^ DPT cells in the blood and liver of patients. **A,** Proportions of each DPT cell subpopulations: CD4^hi^CD8^lo^ (1), CD4^hi^CD8^hi^ (2) and CD4^lo^CD8^hi^ (3) in the blood of each patient (1 dot = 1 patient); percentages lower than 0.05 were plotted as 0. **B,** Same procedure as in (**A**) with LILs. Horizontal lines represent the values of the median for each DPT subpopulation in each group of patients; the three subpopulations of DPT cells correspond to the quadrants set-up in the lower right panel of Figure 1A, (numbers between brackets).

In the blood (Figure 2A), HCV mono-infected patients had a higher percentage (mean ± SD = 3.5±4.7%; median = 1.9%) of CD4^high^CD8^low^ cells (on the left of the graph) than HBV mono-infected (0.7±0.8%; 0.4%) patients, while the proportion of CD4^low^CD8^high^ cells was of the same order of magnitude in both groups: HBV (1.0±0.8%; 0.9%) *vs.* HCV (0.7±0.9%; 0.4%) mono-infected patients. In the liver (Figure 2B), the proportions of CD4^high^CD8^low^ cells were increased in HCV (4.8±5%; 2%) compared to HBV (1.0±1.1%; 0.7%) mono-infected patients, while these HBV (4.1±3.5%; 3.3%) and HCV (3.2±3.2%; 2.4%) patients had a similar proportion of CD4^low^CD8^high^ cells. As with total DPT cells, the proportions were spread within a broad range of values in all groups (Figures 2A and 2B), some of which with very few patients.

**Figure 3:**
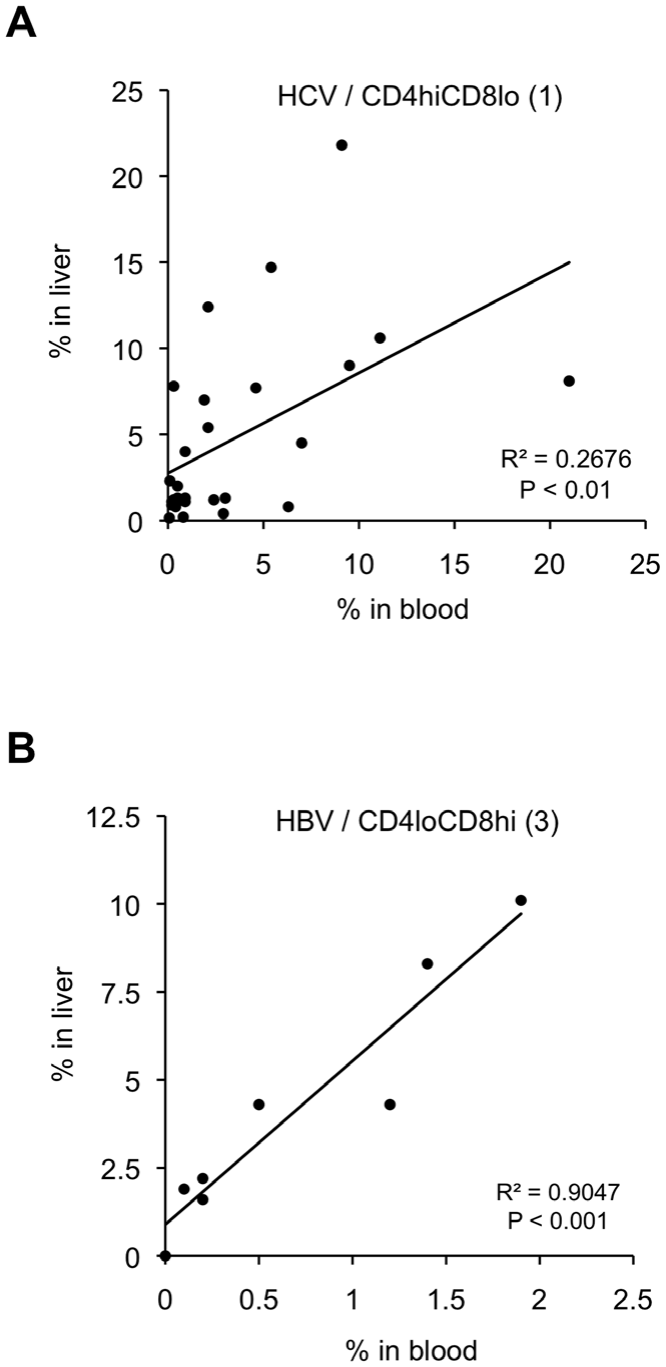
HCV and HBV mono-infected patients display distinct patterns of DPT cells. **A,** Correlation between the proportions of CD4^hi^CD8^lo^ DPT cell subpopulations (1) in blood and liver of HCV mono-infected patients; other subpopulations did not significantly correlate. **B,** Correlation between the proportions of CD4^lo^CD8^hi^ DPT cell subpopulations (3) in blood and liver of HBV mono-infected patients; CD4^hi^CD8^hi^ correlated to the same extent as CD4^lo^CD8^hi^ (respectively 2 and 3) in HBV-infected patients (not shown). Each dot represents the proportions in blood and liver for a patient; significance of Pearson's correlation was tested using a t-test. The numbers between brackets correspond to the quadrants set-up in Figure 1A, lower right panel.

**Figure 4:**
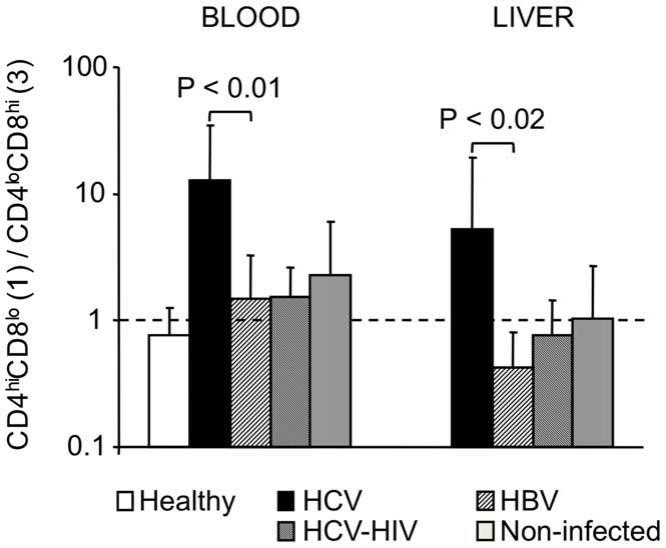
Average ratios of CD4^high^CD8^low^ cells/CD4^low^CD8^high^ cells in the blood and liver of each group of patients. Error bars represent standard deviations. Statistical analysis was performed using a Mann-Whitney test.

We next looked for correlations between the different subpopulations of DPT cells in the blood and liver for each patient, and tested the statistical significance of the difference between HCV and HBV mono-infected patients. The proportions of CD4^high^CD8^low^ DPT cells in blood and liver of HCV mono-infected patients significantly correlated (p<0.01; excluding extreme values increased the significance; Figure 3A), while it was those of CD4^low^CD8^high^ (Figure 3B) and CD4^high^CD8^high^ (not shown) DPT cells that correlated in HBV mono-infected patients (p<0.001, for both); for the other subpopulations of DPT cells, the values in the blood and liver did not significantly correlate in either group. This led us to determine for each patient the ratio of the proportions of CD4^high^CD8^low^ over CD4^low^CD8^high^ DPT cells in the blood and the liver. In average this ratio was at least five times higher in the blood (p<0.01) and the liver (p<0.02) of HCV mono-infected patients compared to the other groups (Figure 4).

**Figure 5:**
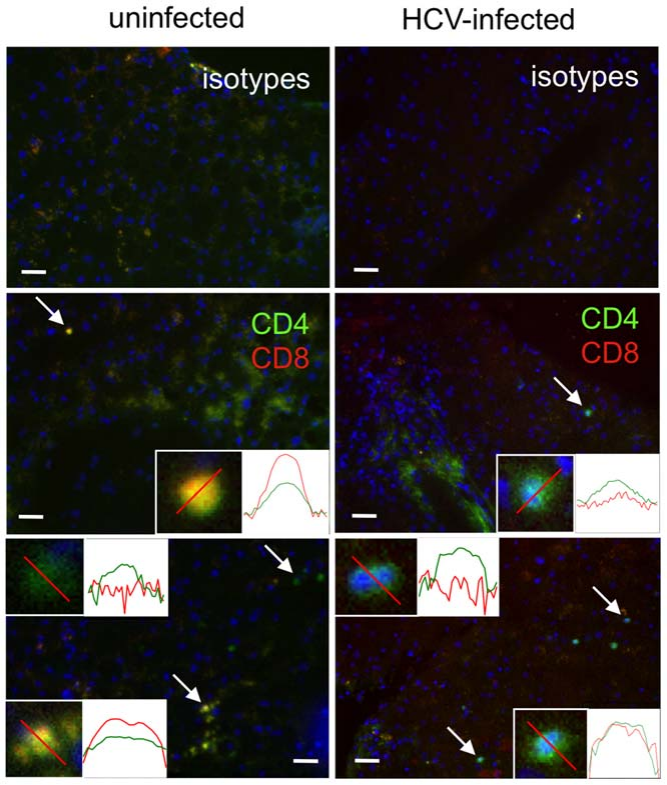
*In situ* study of LILs by immuno-fluorescence in liver sections of uninfected and HCV-infected patients. Results obtained with liver biopsies from an uninfected (left panels) and an HCV-infected (right panels) patient. 10-µm serial sections were labeled with CD4 and CD8 antibodies (middle and bottom panels) or isotype controls (top panels). Scale bar: 100 µm. Right Insets: relative fluorescence intensities along the red bars in the left insets plotted on a semi-logarithmic scale (0–100), for the red (CD8) and green (CD4) light channels.

The presence of DPT cells was verified *in situ* in snap frozen liver biopsies. CD4 (green) and CD8 (red) cell markers were studied side-by-side in one HCV-infected (Figure 5, right panels) and one non-infected (Figure 5, left panels) patient. Cells with either signal, or both, were detected; their relative quantifications in individual cells (insets; semi log scale) suggested that more DPT cells displayed a greater red (CD8) than green (CD4) signal in non-infected than in HCV-infected liver (Figure 5, middle and lower left panels; yellowish cells). In contrast, DPT cells with equivalent green and red signals were found at a higher frequency in HCV-infected than in non-infected liver. The distinct nature of the signals measured by fluorescence microscopy (staining density in whole cells) and by flow cytometry (integration of staining at the cell surface) precludes the direct comparison of signal intensities between liver sections and extracted LILs, respectively. Nevertheless, the *in situ* results indirectly confirm the existence of distinct patterns of DPT cells in HCV-infected and uninfected patients.

### Intra-hepatic DPT cells in chronically HCV-infected patients are effector/memory T cells and express CD1a

To determine the degree of differentiation and activation of DPT cells, we performed additional phenotypic analyses in six patients for whom enough cells had been harvested. In the blood of chronically HCV-infected patients, while CD1a, CD25, CD27, CD28 or CD62L markers were not detected in most DPT cells, about 30% of them expressed the activation marker CD38. The proportions of CCR7^+^ cells were similar to those in HCV-uninfected individuals; more than half of the CD4^low^CD8^high^ DPT cells expressed CCR7, a marker of naïve and memory T cells, which was detected much less often in CD4^high^CD8^low^ DPT cells. Contrasting with these results, DPT cell subpopulations were more heterogeneous in the liver; 53% of CD4^low^CD8^high^, 59% of CD4^high^CD8^low^ and 75% of CD4^high^CD8^high^ DPT cells of chronically HCV-infected patients expressed CD45RO, CD62L and CCR7, typical of central memory phenotype, while 23 to 46% of them were minus either for CCR7 or CD62L, i.e. corresponded to effector memory T cells. Most intra-hepatic CD4^low^CD8^high^ and CD4^high^CD8^low^ DPT cells expressed the marker CD25, and had lost the expression of CD27 or CD28, or both, suggesting they were differentiated cells. CD4^high^CD8^high^ DPT cells were more heterogeneous since about half of them retained CD27 and CD28 expressions. Strikingly, 80 to 90% of intra-hepatic DPT cells expressed CD1a, a marker of T cell development within the thymus [20], at odd with its lack of detection in circulating DPT cells.

### Experimental evidence for the direct regulation of DPT cell phenotype by HCV in human liver

**Figure 6:**
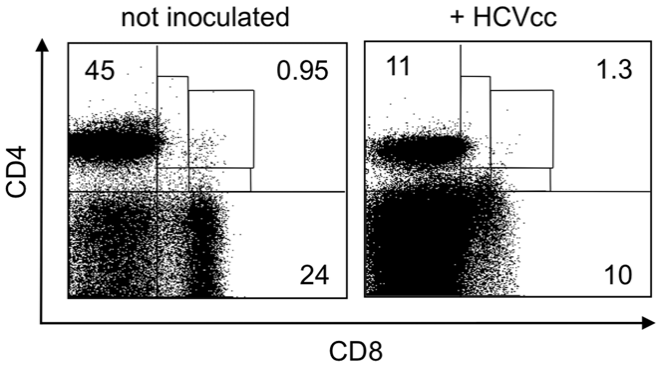
Flow cytometry analysis of DPT cell subpopulations extracted from cultured human liver slices. Human liver slices (350 µm thick) were obtained from the surgery of uninfected patients and inoculated (MOI = 0.1), or not, with HCVcc of the strain JFH-1 (genotype 2a) produced in the HCV-permissive Huh-7.5 cell line; after 5 days *ex vivo*, LILs were isolated from the slices and analyzed as in Figure 1.

We have obtained evidence that naïve human liver slices can be infected *ex vivo* by infectious HCV particles (HCVcc) produced in permissive hepatic cell lines [21]. We reasoned that if chronic inflammation was underlying the DPT cell phenotype observed in HCV-infected patients, no major change should occur after only a few days. We therefore prepared uninfected human liver slices and inoculated them with HCVcc [16]; after five days, LILs were extracted from the slices and harvested by flow cytometry using the same procedure as for liver biopsies (cf. Materials and Methods). Surprisingly, upon infection the LILs were more numerous than in control slices (Figure 6), although this could result from either cell proliferation or increased cell survival. Interestingly, we found that HCVcc induced T cell proliferation (measured by staining with CFDA-SE [22]) in liver slices, but not in immune cells previously extracted from liver (not shown), in line with the former hypothesis. Moreover, the proportions of CD8^+^ single-positive T cells decreased as well as both CD4 and CD8 expression levels, suggesting a direct effect of the presence of the virus on the differentiation of intra-hepatic lymphocytes. Finally, the proportion of DPT cells was slightly higher, and CD4^high^CD8^low^ cells were more frequently observed, whereas CD4^low^CD8^high^ and CD4^high^CD8^high^ cells almost disappeared.

## Discussion

The goal of our study was to gain some insight regarding the origin of extra-thymic DPT cells during HCV infections. Intra-hepatic and circulating DPT cells were comprised of populations of activated, central and effector memory cells, with heterogeneous differentiation patterns, in accordance with results in the liver of a chimpanzee at the acute phase of HCV infection [9]. Discrepancies in the proportions of circulating DPT cells could relate to methodological differences and/or patient samples (e.g. stages of HCV infection). With comparable criteria, the chronically HCV-infected patients of the present study had heterogeneous proportions of DPT cells, and displayed a different pattern than chronically HBV-infected patients.

Chronic inflammation of the liver is unlikely the sole reason underlying enhanced proportions of DPT cells, since the latter were detected in only half of the patients chronically infected by HCV, and even less often in those infected by HBV. HCV and HBV infections did not yield the same patterns of DPT cells, suggesting the existence of viral specificities. Although the proportion CD4^high^CD8^low^ cells is increased in HIV-infected patients [18], it was not obvious in our patients that HCV co-infection further enhanced their frequency, or conversely; instead, HIV-HCV co-infected patients displayed proportions of CD4^low^CD8^high^ cells as high as in HBV mono-infected patients. Therefore, the mechanisms of induction of CD4^high^CD8^low^ cells by HCV and HIV are likely distinct, pointing again at virus specificities.

Experimentally, HCV infection changed the phenotype of intra-hepatic DPT cells, *ex vivo*. Thus, the presence of HCVcc modified the patterns of T and DPT cells in liver slices. Such effect was not observed with extracted hepatic immune cells and, therefore, likely resulted from hepatocyte infection after inoculation of the slices with HCVcc. The change having taken place within five days, the peculiar pattern of DPT cells observed in patients hence cannot only be explained by chronic infection. Alternately, albeit not exclusively, the pattern of intra-hepatic DPT cells could result from direct effects of HCV on other local/resident immune cells [23], or have more indirect causes.

LILs of patients chronically infected with HCV include a population of DPT cells expressing CD1a^+^ cells. However, unlike naïve DPT cells that escape from the thymus [8], [24], circulating DPT cells in HCV patients had an activated, differentiated memory phenotype. In addition, they did not express CD1a; even if recruited to the liver, these cells were unlikely to re-express this marker, since in the periphery it is generally expressed onto lipid antigen-presenting cells [25]. Chronic immune processes generally deplete the thymus [26], [27]; for DPT cells to originate from the thymus, at least in some patients, HCV infection had to overcome the depletion by re-inducing T cell production.

Thus, the presence in the liver of CD1a^+^ cells expressing both CD4 and CD8 co-receptors could result from the accelerated recruitment of precursors from the bone marrow followed by their rapid development towards a T cell phenotype in the thymus. It could be a compensatory mechanism for the loss of terminally differentiated T lymphocytes resulting from the chronic infection or, more directly, viral factors. Indeed, the existence of a plasticity of the thymus has recently been recognized [27], [28] and, here, could involve the enhanced production of IL-7 in the liver [28], [29]. According to this hypothesis, HCV infection would induce the rapid differentiation and/or relocation of CD1a^+^ DPT cells in the liver. Consistent with a correlation between predominant intra-hepatic and circulating subpopulations, as well as progression of their phenotype between the liver and the blood compartments, DPT cells would be re-circulating from the liver after their local activation, like other T cells [30].
